# Older Adults with Autism Spectrum Disorders in Sweden: A Register Study of Diagnoses, Psychiatric Care Utilization and Psychotropic Medication of 601 Individuals

**DOI:** 10.1007/s10803-018-3567-0

**Published:** 2018-04-16

**Authors:** Lena Nylander, Anna Axmon, Petra Björne, Gerd Ahlström, Christopher Gillberg

**Affiliations:** 1 Department of Clinical Sciences/Psychiatry, Lund University, 221 00 Lund, Sweden; 2 Division of Occupational and Environmental Medicine, Lund University, 221 00 Lund, Sweden; 3 Research and Development Unit, City Office, City of Malmö, 205 80 Malmö, Sweden; 4 Department of Health Sciences, Lund University, 221 00 Lund, Sweden; 5 Gillberg Neuropsychiatry Centre, Institute of Neuroscience and Physiology, Sahlgrenska Academy, Gothenburg University, Gothenburg, 411 19 Sweden; 6 VUB-teamet Psykiatriska Kliniken, Baravägen 1, 221 85 Lund, Sweden

**Keywords:** Older adults, Autism spectrum disorders, Psychiatry

## Abstract

In a Swedish sample of persons eligible for disability services and aged 55 years or older in 2012, persons (n = 601) with autism spectrum disorder diagnoses registered in specialist care were identified. Register data concerning diagnoses of other psychiatric disorders, psychiatric care, and psychiatric medication were reviewed. More than 60% had been in contact with psychiatric care. The majority had no intellectual disability (ID) diagnosis recorded during the study period. Apart from ID, affective disorders, anxiety and psychotic disorders were most commonly registered; alcohol/substance abuse disorders were uncommon. Psychotropic drug prescriptions were very common, especially in the ID group. Professionals need awareness of this vulnerable group; studies concerning their life circumstances and service requirements should be conducted.

## Introduction

Autism spectrum disorders (ASDs) are often presumed to be life-long disabilities. There are several follow-up studies showing that these disorders, which in all cases are present at an early age, are relatively stable from childhood to adolescence (Nordin and Gillberg 1998; Szatmari et al. 2009), and, especially if accompanied by intellectual disability (ID), into adulthood (Billstedt 2007). In a review of 23 follow-up studies, Howlin and Moss (Howlin and Moss 2012) found that as adults, many individuals with ASD were significantly disadvantaged in several ways, including physical and mental health. Although two studies of the same Swedish cohort with normal IQ ASD (n = 47) showed that the number who met diagnostic criteria for any ASD slowly diminished (Cederlund et al. 2008; Helles et al. 2015), 76% still had the number of symptoms required for an ASD diagnosis even in the follow-up study at mean age 30 years.

Only two of the 23 studies in the Howlin and Moss review included any individuals older than 50 years, and most ASD research today is still on children (Howlin 2008; Howlin and Moss 2012; Mukaetova-Ladinska et al. 2012). Thus, very little is known about ASD in people older than around 50 years (Happé and Charlton 2011; Perkins and Berkman 2012; Barber 2015; Lai and Baron-Cohen 2015; Bennett 2016) and only recently has work been commenced in this area. Among other things, it is not known how many older individuals have diagnosed ASDs, how ASD is expressed in older people, or if coexisting physical and mental disorders are common. In recent years, there have been some studies on this subject, primarily in the UK (James et al. 2006), the US (Kats et al. 2013), and the Netherlands (van Niekerk et al. 2010; Geurts and Vissers 2011; van Heijst and Geurts 2014; Geurts et al. 2016; Lever and Geurts 2016). However, the knowledge concerning this group is still very limited, both in Sweden and in most other countries.

A previous study by the research team (Axmon et al. 2016) examined use of psychiatric services in a sample of older adults (n = 7936, mean age 53, age range 44–85) from the general population, and found that only 6.4% had any registered visit to psychiatric care (inpatient or outpatient) during the 11-year period that the study comprised. Older people with ID had higher inpatient and outpatient care utilisation (OR 3.59).

In this study we focused on ASD, more specifically in the older adult Swedish population eligible for municipal services. The aim was to investigate the prevalence of individuals with ASD diagnoses from specialist care, with and without concurrent diagnoses of ID of varying severity. The term “specialist care” refers to all medical clinics that are not considered primary care, or general practice. The aims included investigation of the pattern of coexistent psychiatric diagnoses and the utilisation of psychiatric care and psychotropic medication among any individuals found to have ASD diagnoses.

## Materials and Methods

The present study is a nation-wide register study conducted in Sweden and comprising the years 2002–2012, with the exception of data on prescribed drugs which have been obtained from 2006 only.

### National Registers Used in the Study

In Sweden, individuals with permanent and considerable functional impairment can apply for municipal services according to the Act Concerning Support and Service for Persons with Certain Functional Impairments (Swedish abbreviation: LSS; 1993:387). The Act covers individuals who can be categorised as belonging to one of three specific groups. Group 1 consists of people who have been diagnosed with ID and/or ASD, and are thus in need of services. Group 2 consists of people with cognitive disability after sustaining brain injury/damage in adulthood, while Group 3 consists of people in need of support due to other permanent and considerable disabilities that hinder their ability to function in daily life. Group 1 is thus the only group that is defined mainly by diagnosis.

Individuals provided with support from the municipality under the umbrella of this act are included in the LSS-register as well as data on which type of support they received. This register is maintained by the Swedish National Board of Health and Welfare and does not contain information on individuals’ diagnoses, only on their LSS-group classification. The register contains only those who, after their own application, have been found to meet criteria to be given support. Thus, not everyone diagnosed with ASD or even ID can be expected to be registered, which means that a number of people with ASD, especially people with normal IQ, may not be included in the study group.

The Swedish National Board of Health and Welfare also maintains The Swedish National Patient Register (NPR). The NPR contains information on all individuals using in- and outpatient specialist medical care in Sweden. It contains no information about visits to primary health care. Specialist psychiatric care includes all psychiatric inpatient care, as well as any outpatient care considered to require resources above the primary care level. Registration in the NPR is made at the date of discharge for inpatient care, and at the date of the visit for outpatient care. Every time a patient is registered in this way, one primary and up to 21 secondary diagnoses are listed. Diagnoses are coded according to the 10th revision of the International Classification of Disease (ICD-10) (WHO 1993). Diseases and/or conditions that are not the focus of the care episode may or may not be registered as secondary diagnoses, depending on their relevance to the circumstances, local procedures, or the physician’s own preferences. It is not possible to find information in the NPR on where or when any diagnosis was first made. In the ICD-10, the term “mental retardation” is used rather than “intellectual disability”. However, as “intellectual disability”/ID is the currently preferred term (Schalock et al. 2007), we will use this henceforth.

Also the Swedish Prescribed Drug Register (PDR) is kept at the Swedish National Board of Health and Welfare, and contains information from July 2005 onwards on dispensed prescribed drugs (Wettermark et al. 2007).

### Study Population

Through the LSS register, we identified 7936 persons (3609 women and 4327 men) in Sweden who met the following criteria: (1) they were 55 years or older on December 31st, 2012, (2) they belonged to LSS-group 1 (i.e. had been diagnosed with ID and/or ASD), (3) they had received at least one service according to LSS during 2012, and (4) they were alive at the end of 2012. As outcome data was obtained for the time period 2002–2012, information was collected for persons aged 44 years and above. The mean age on December 31, 2012, was 64 years (range 55–96 years).

All individuals who, during the period examined (2002–2012), had a registered diagnosis of any ASD—defined as an ICD-10 code for pervasive developmental disorder (PDD), F84.0-84.9 (n = 606, 7.63%)—were extracted using the diagnoses registered in the NPR. Five of these 606 were excluded: four women with Rett’s syndrome, F84.2, as single diagnosis and one man with other childhood disintegrative disorder (F84.3), leaving 601 persons (385/64% males and 216/36% females) in the study group. However, several individuals (103; 17%) had more than one F84 diagnosis, two of which had as many as four different diagnoses. Therefore, diagnoses were ranked in order to classify each person with only one F84 diagnosis. The chosen rank order was childhood autism (F84.0), Asperger’s syndrome (F84.5), atypical autism (F84.1), other PDD (F84.8) and PDD, unspecified (F84.9), based on the assumption that childhood autism and Asperger's syndrome, respectively, refer to more “typical” variants of ASD than the following terms. Only three individuals in the sample had been registered with both childhood autism (F84.0) and Asperger’s syndrome (F84.5) diagnoses. Since 1419 persons in the LSS group were not registered in the NPR, i. e. did not visit inpatient or outpatient specialist care during the period examined, we have no information regarding their possible ASD diagnoses. The 601 remaining individuals with registered ASD diagnoses will be referred to below as the ASD group. All persons in the ASD group had at least one point of registration in the NPR during 2002–2012, meaning that they had been in contact with specialist medical care and that ASD had been observed by the physician.

Data concerning gender, other psychiatric diagnoses, psychiatric care utilisation and psychotropic medication were reviewed, using data from the NPR (2002–2012) and PDR (2006–2012).

In the NPR, the type of clinic where each visit is made is registered based on codes determined by the Swedish National Board of Health and Welfare. For this study, the nine different types of adult psychiatric services were collapsed into three groups, namely “general adult psychiatric service” (also encompassing “psychiatric nursing home”, “geropsychiatric service”, “specialised psychiatric care” and “psychiatric rehabilitation”), “forensic psychiatric care on regional level” and “substance dependency treatment” (also including “alcohol dependency care” and “toxicomania care”). Registrations in any type of psychiatric clinic were considered in total (any registration), as well as inpatient and outpatient registrations separately. We also calculated the mean and median number of days in inpatient care for each of the ASD groups.

Three patient records were registered as “psychiatric care for children and adolescents” and four records were missing information on type of clinic and were therefore excluded in this study, as were 73 records which were duplicates with respect to person, clinic and date.

A number of individuals with ASD diagnoses also had registered ID (ICD-10 code F70-79) diagnoses during the period examined. In some cases a person had been diagnosed with ID of different severities on different visits. We have chosen a rank order where a specified ID takes precedence over an unspecified ID, and the most severe ID diagnosis takes precedence over the milder variants. Concomitant psychiatric diagnoses other than ID were categorised in nine groups: ADHD (F90), psychotic disorders (F2), affective disorders (including bipolar) (F3), anxiety disorders (F40-42), personality disorders (F60-61), alcohol/substance use related disorders (F1 except F10.7A), dementia (F00-03, F10.7A, F05.1) and other psychiatric disorders (all other registered F diagnoses not belonging to any of the above-mentioned groups).

From the PDR, information was collected concerning certain psychotropic drugs, grouped according to the Anatomical Therapeutic Chemical Classification (ATC-code). The drug categories chosen were antipsychotics (ATC-code N05A), anxiolytics (N05B), hypnotics and sedatives (N05C) and antidepressants (N06A).

**Fig. 1:**
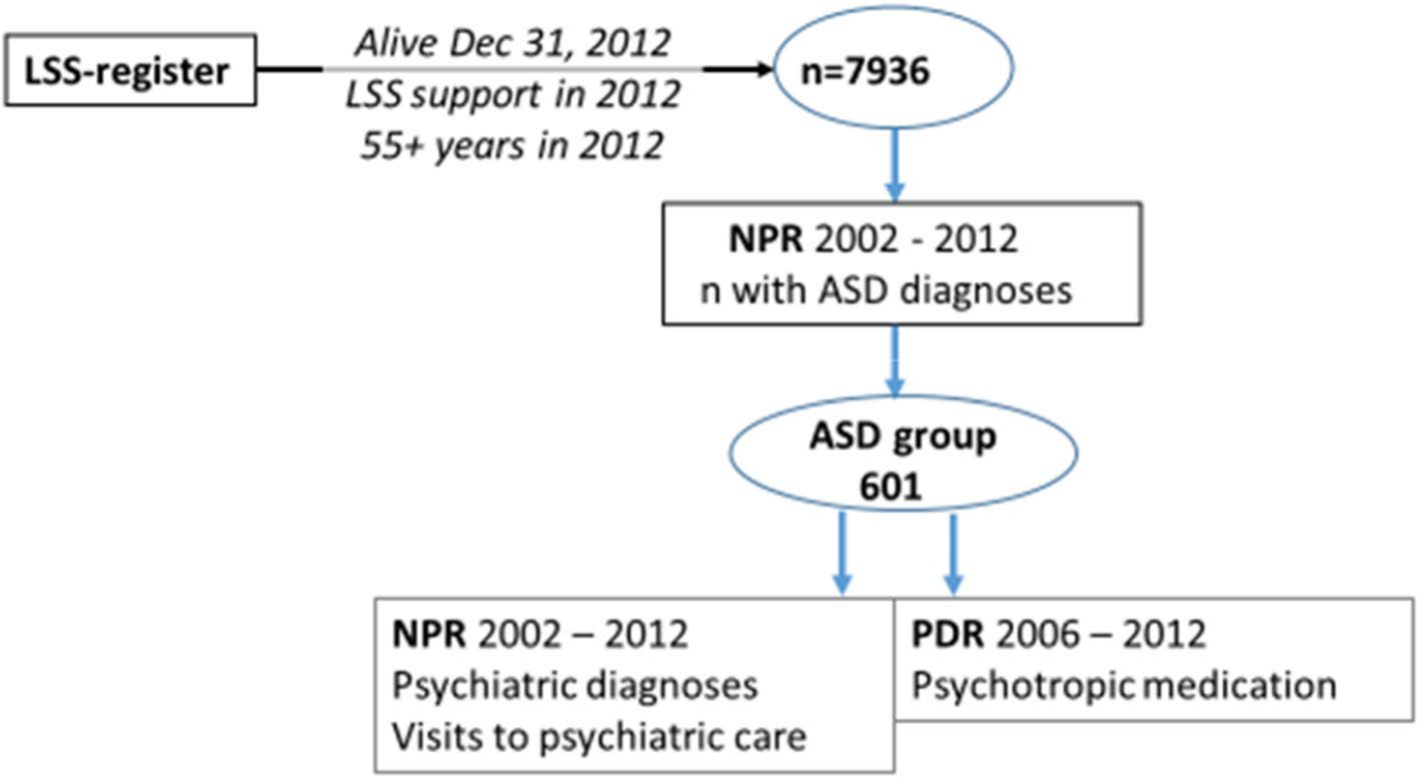
Data catchment procedure

Figure 1 shows the procedure of data inclusion.

### Ethics Approval and Consent to Participate

The study was approved by the Regional Ethical Review Board. This study is part of a larger project, which used anonymised datasets drawn from four—in the present study three—official national registries maintained by the National Board of Health and Welfare and Statistics Sweden. The National Board of Health and Welfare and Statistics Sweden provided separate secrecy reviews in 2014 before access to the data. Due to the requirement for anonymised data, individuals could not be asked for consent to participate; instead the reverse principle was applied, with active refusal of participation required to avoid inclusion in the study. Information about the planned study was published in the national newspaper ‘Dagens Nyheter’ and one version was easy-to-read text for the UNIK, the magazine of the Swedish National Association for Persons with Intellectual Disability (print run of 22,000 copies). The target audience for the UNIK magazine are mainly members (people with ID) and their families. The advertisement presented the study and contained information on how to contact the research manager by phone, email or mail to opt out of the study. The research manager was responsible for contacting the national registries to ensure that those not wanting to participate were excluded before the registries provided any data to the research manager. There were no refusals to participate.

### Funding

This work was funded by the Swedish Research Council for Health, Working Life and Welfare. The funding agency had no role in the design, analysis, and interpretation of this study. The requirement from the national fund is only Open Access publishing.

## Results

**Fig. 2:**
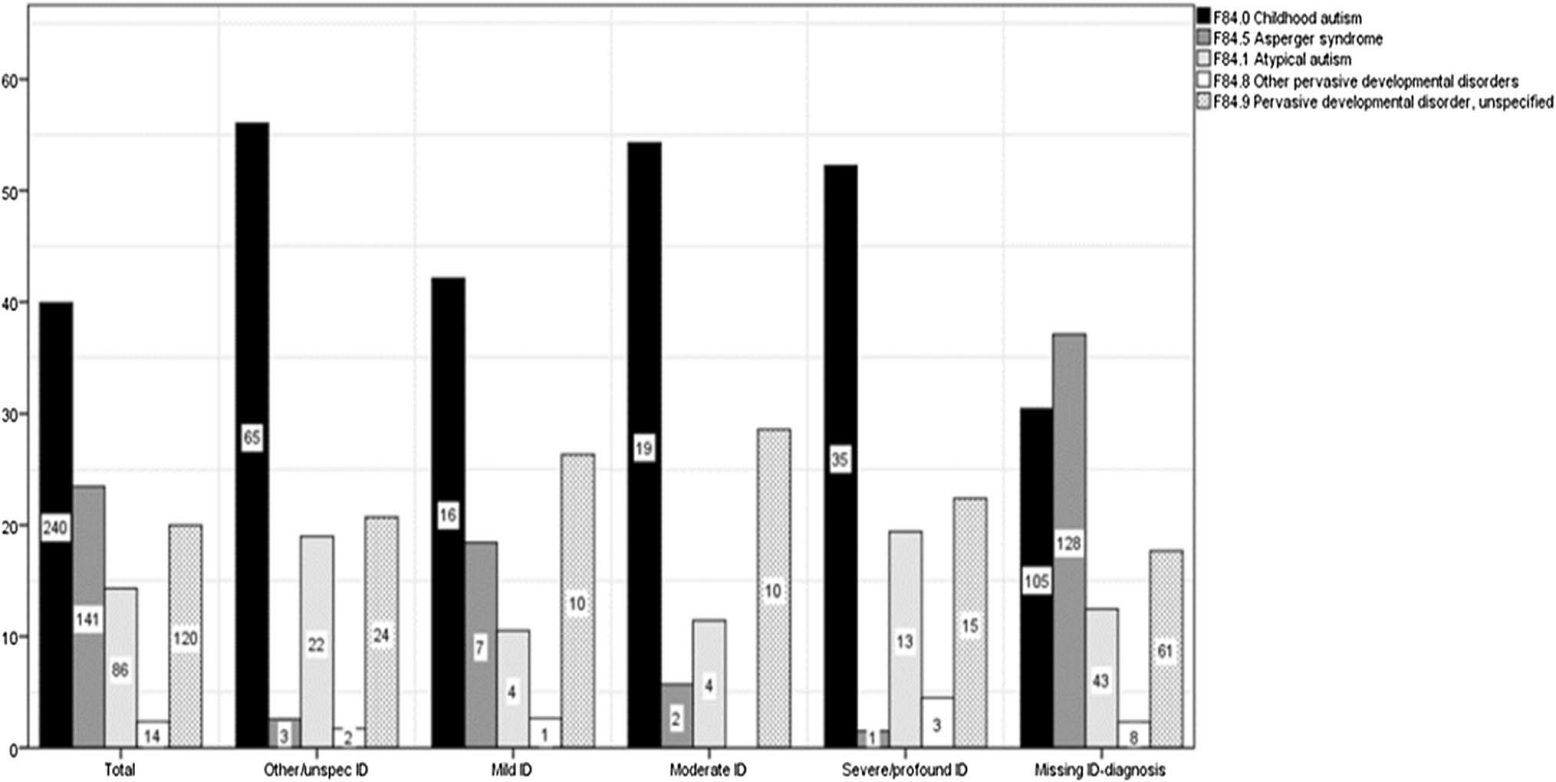
Percentage of different ASD-diagnoses within each ID category (y-axis), with each bar marked with the actual number of people

Figure 2 shows gender and birth year distribution for the five categories of ASD in the study group (n = 601). The age range in 2012 was 55 to 96 years. Childhood autism (F84.0) is the most common diagnosis irrespective of gender or age. Asperger’s syndrome (F84.5) is more common in persons born 1950 or later than in older persons. Slightly more women than men had atypical/unspecified ASD diagnoses, which was also seen in the patients born before rather than after 1950.

Table 1 shows ASD categories in relation to concomitant ID (F7) diagnoses. There were no ID diagnoses registered for 345 persons (57%). Mild ID (6%) was less common than severe/profound (11%), and most common was other/unspecified ID (19%). Thirteen individuals (9%) of the Asperger’s syndrome group—commonly defined as ASD in the presence of a normal or high intellectual level and useful language—were assigned ID diagnoses of different severities, one of them even classified as severe/profound. Forty-eight individuals (42%) in the group with other/unspecified ID had atypical/other/unspecified ASD diagnoses, and 19% of the ASD group had been assigned other/unspecified ID diagnoses.

**Table 1:** Number and percentage of individuals with ASD diagnoses within each ID category

	Mild ID	Moderate ID	Severe/profound ID	Other/unspecified ID	No ID diagnosis
	n (%)	n (%)	n (%)	n (%)	n (%)
F84.0 childhood autism n = 240	16 (6.7)	19 (7.9)	35 (14.6)	65 (27.1)	105 (43.8)
F84.5 Asperger syndrome n = 141	7 (5.0)	2 (1.4)	1 (0.7%)	3 (2.1)	128 (90.8)
F84.1 atypical autism n = 86	4 (4.7)	4 (4.7)	13 (15.1)	22 (25.6)	43 (50.0)
F84.8 other pervasive developmental disorders n = 14	1 (7.1)	0 (0.0)	3 (21.4)	2 (14.3)	8 (57.1)
F84.9 pervasive developmental disorder, unspecified n = 120	10 (8.3)	10 (8.3)	15 (12.5)	24 (20.0)	61 (50.8)
Total n = 601	38 (6.3)	35 (5.8)	67 (11.0)	116 (19.3)	345 (57.4)

Of the 601 persons with ASD, 86 had two different ASD diagnoses, 15 had three different diagnoses and two had four different diagnoses.

**Fig. 3:**
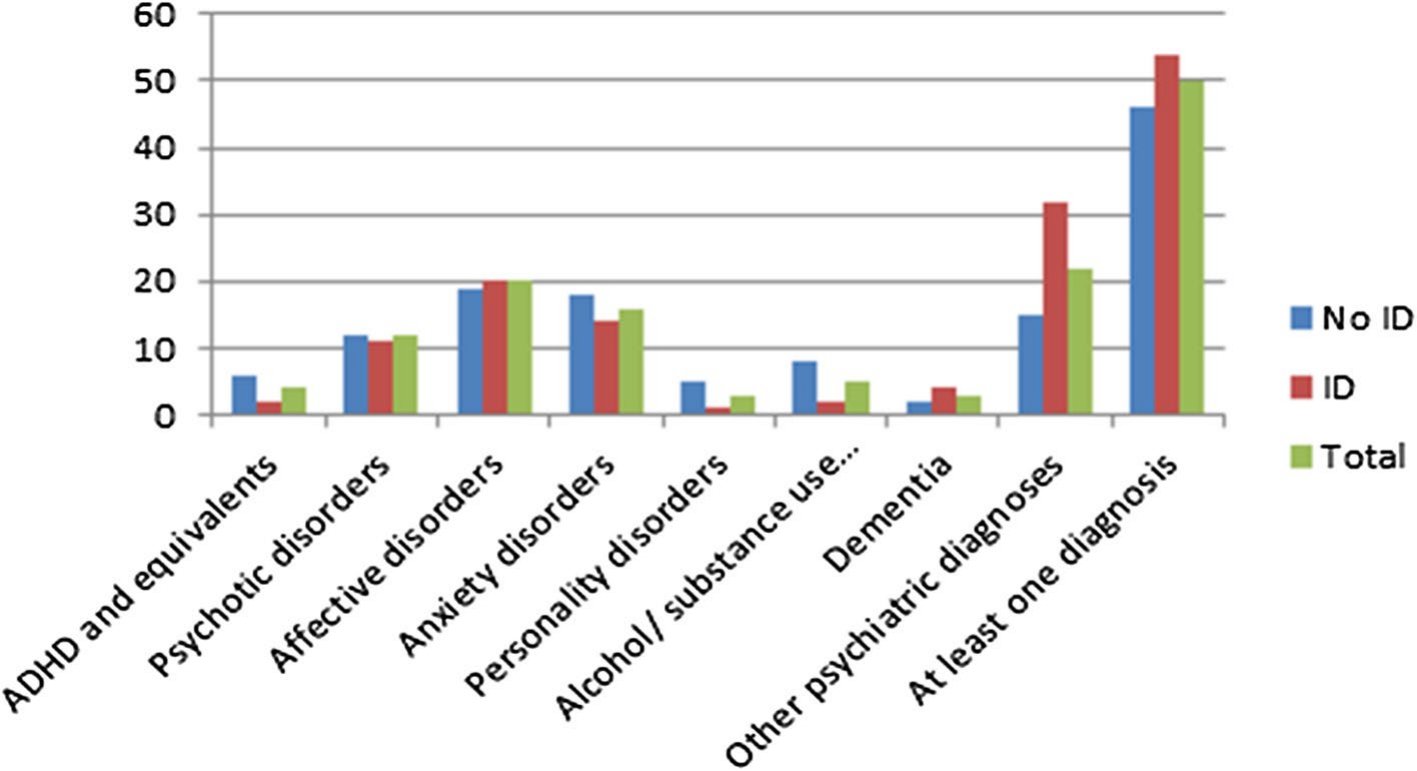
Psychiatric diagnoses in people with/without ID and in the whole

Figure 3 shows that, apart from the heterogeneous group “other psychiatric disorders”, affective disorders were the most commonly registered conditions, followed by anxiety and psychotic disorders. This pattern is fairly consistent across the ASD categories. The group with no ID dominates among those with registered psychiatric diagnoses. Most common in this group were affective and anxiety disorders. People with ASD and ID have also often been diagnosed with psychiatric disorders, especially disorders in the “other” group.

A small number of patients had been diagnosed with ADHD. Similar to alcohol and/or substance use-related disorders, ADHD was diagnosed mainly in the group without ID.

Table 2 shows the number of persons with psychiatric care utilisation as well as the number of psychiatric care visits. Number of visits (out-patient as well as in-patient) to different categories of adult psychiatric care is shown, as well as number of days as psychiatric inpatient for the five ASD categories. Almost all visits are made in clinics belonging to the category “general adult psychiatric care”. Visits to clinics giving forensic psychiatric care had been made by people diagnosed with Asperger’s syndrome (F84.5) and PDD, unspecified (F84.9). Only individuals with an Asperger’s syndrome diagnosis, had made visits to substance dependency treatment clinics. The group with Asperger’s syndrome had the highest number of people who had spent time as psychiatric inpatients. 220 (37%) of the 601 persons had not been in psychiatric care during the period examined. Their ASD diagnoses have thus been registered in somatic specialist care.

**Table 2:** Psychiatric health care utilization

	Number of visits to different categories of psychiatric clinics^a^			Number of people with different psychiatric health care utilization			Total number of days in psychiatric inpatient care	
	General adult psychiatric service	Forensic psychiatric care on regional level	Substance depen-dency treatment	No psychiatric care	Psychiatric out- but not inpatient care	Psychiatric inpatient care		
	n (%)	n (%)	n (%)	n (%)	n (%)	n (%)	Mean	Median
F84.0 childhood autism (n = 240)	823 (100)	0 (0)	0 (0)	108 (45)	84 (35)	48 (20)	43	15
F84.5 Asperger syndrome (n = 141)	1362 (90)	27 (2)	117 (8)	15 (11)	66 (47)	60 (43)	197	65
F84.1 atypical autism (n = 86)	280 (100)	0 (0)	0 (0)	37 (43)	37 (43)	12 (14)	33	18
F84.8 other pervasive developmental disorders (n = 14)	24 (100)	0 (0)	0 (0)	7 (50)	4 (29)	3 (21)	2	2
F84.9 pervasive developmental disorder, unspec (n = 120)	368 (97)	13 (3)	0 (0)	53 (44)	39 (33)	28 (23)	105	25

^a^One person may contribute with more than one visit

Table 3 shows number, percentages and the OR for using any specialist psychiatric care for the ASD subgroups, ID versus no ID, gender and age group, respectively. The OR for people with Asperger’s syndrome to have contact with specialist psychiatry was almost seven compared to people with childhood autism. The OR was also elevated for people with ID compared to no ID, for males and for younger versus older people.

**Table 3:** Any psychiatric care versus no psychiatric care

	Any psychiatric care n (%)	No psychiatric care n (%)	OR for any psychiatric care versus no psychiatric care	95% CI
ASD subgroup				
F84.0 childhood autism	132 (55)	108 (45)	Ref	
F84.5 Asperger syndrome	126 (89)	15 (11)	6.87	3.80–12.43
F84.1 atypical autism	49 (57)	37 (43)	1.08	0.66–1.78
F84.8 other pervasive developmental disorder	7 (50)	7 (50)	0.82	0.28–2.41
F84.9 pervasive developmental disorder, unspecified	67 (56)	53 (44)	1.03	0.67–1.61
ID/no ID				
No ID	193 (56)	152 (44)	Ref	
ID	188 (73)	68 (27)	2.18	1.54–3.09
Gender				
Women	128 (59)	88 (41)	Ref	
Men	253 (66)	132 (34)	1.32	0.94–1.86
Age group				
–1944	54 (50)	53 (50)	Ref	
1945–1949	93 (61)	60 (39)	1.52	0.92–2.51
1950–1954	128 (65)	68 (35)	1.85	1.14–2.99
1955–	106 (73)	39 (27)	2.67	1.57–4.52

Table 4 shows the number and percentage of people, categorised as those with and without a registered ID diagnosis, with at least one prescription of psychotropic drugs. The drugs investigated were categorised as neuroleptics (N05A), anxiolytics (N05B), hypnotics and sedatives (N05C) and antidepressants (N06A). The table also shows the number and percentage of people prescribed with more than one category of the above mentioned drugs.

**Table 4:** Individuals with ASD diagnoses and at least one prescription of psychotropic drugs (antipsychotics, anxiolytics, hypnotics and sedatives, antidepressants)

	No ID diagnosis (n = 345)	ID diagnosis (n = 256)
	n (%)	n (%)
At least one prescription of		
Antipsychotics	217 (63)	214 (84)
Anxiolytics	204 (59)	198 (77)
Hypnotics and sedatives	172 (50)	145 (57)
Antidepressants	171 (50)	152 (59)
No of psychotropic drugs		
0	46 (13)	12 (5)
1	65 (19)	26 (10)
2	69 (20)	49 (19)
3	99 (29)	91 (36)
4	66 (19)	78 (30)

## Discussion

To our knowledge, this register study describes the largest group of older adults with ASD diagnoses that has ever been studied. Several authors (e. g. Happé and Charlton 2011; Perkins and Berkman 2012; Bennett 2016) have described the need for research and data on older people with ASD, and our study provides knowledge concerning psychiatric diagnoses, psychiatric care and psychotropic medication.

The most common ASD diagnosis in the group was childhood autism (F84.0), often regarded as the most severe form of ASD. This may be due to diagnostic traditions—with only one available autism diagnosis instead of a spectrum—when the persons were young and the autism diagnosis was first made, and/or the selection of the group being based on their need for extensive help. In the younger subgroup (born after 1950), Asperger’s syndrome (F84.5) was a more common diagnosis than in the older part of the group. Since we have no way of knowing when any diagnosis in the group was first made, we have no explanation for this, other than that the Asperger’s syndrome diagnosis was first listed in ICD-10 (WHO 1993) and that it has slowly become more well-known and used in the past 10–15 years. However, since the more recent diagnostic manual DSM-5 (American Psychiatric Association 2013) lists only one label, autism spectrum disorder (ASD), it is to be expected that other diagnoses, e.g. childhood autism or Asperger’s syndrome, will be less used in the future.

Studies from recent years have shown that ASD may be more difficult to diagnose in females than in males (Mandy et al. 2012; Frazier et al. 2014) and this is indicated by our study as well, where there was a tendency towards other or unspecified ASD being more common among women than in men. The same tendency was seen in the older ASD group as compared with the younger one. However, tendencies in this study must be regarded with caution, since no measures of statistical significance are included.

The majority of the group had no registered ID diagnosis. This could be due either to under registration of ID, or to ASD being diagnosed in increasing numbers in individuals with intellectual functioning in the normal range, including in older adults. Surprisingly, mild ID was less common than severe ID in the ASD group, and most common was other/unspecified ID. This is probably an effect of the ID diagnoses being registered in connection with visits in the care system on account of symptoms of illness rather than after a cognitive assessment; moreover, the physicians in question may have had registered information provided to them by care staff from social services in the event that he/she did not have access to earlier evaluations. If care staff found information about ASD more pertinent, mild ID may have stayed unregistered. Furthermore, severe/profound ID may be more conspicuous during a medical examination. Also, it is more likely that individuals with more severe impairments and pervasive needs are registered in the LSS register. This register, as mentioned above, can thus not be expected to include all people with ASD, which is a limitation to this study.

As in general adult psychiatry, apart from the heterogeneous group “other psychiatric disorders”, affective disorders were the most commonly diagnosed concomitant psychiatric disorders, followed by anxiety and psychotic disorders, in the total ASD sample. Patients with childhood autism (F84.0) and ID had more “other” psychiatric disorders than the other groups, which may reflect diagnostic difficulties with patients who often have very low communicative skills. Affective and anxiety disorders were often diagnosed in patients with Asperger’s syndrome (F84.5) without ID, which is in concordance with other studies (Lever and Geurts 2016). Alcohol and/or substance use-related disorders were diagnosed mainly in the group without ID, probably reflecting the fact that the more disabled persons had very limited access to the pathogens in question. Only patients with Asperger’s syndrome diagnoses had been visiting specialist clinics for these disorders, which, according to clinical experience, may also be underdiagnosed and thus undertreated in persons with ID.

Our results are, to a great extent, in line with the earlier studies on adults with ASD, which have shown that concomitant psychiatric symptoms and diagnoses are common (Hutton et al. 2008; Hofvander et al. 2009; Buck et al. 2014; Hirvikoski et al. 2016; Helles et al. 2015), also when compared to other psychiatrically referred adults (Joshi et al. 2013). Patients with Asperger’s syndrome diagnoses, F84.5 and no ID make up the majority among those with registered psychiatric diagnoses. Affective and anxiety disorders were the most common concurrent disorders, as was the case in for example the younger normal IQ sample studied by Hofvander et al. (2009). Similarly, Hutton et al. (2008) also reported that mood disorders were the most frequent new-onset psychiatric disorders in their study group. In contrast to the latter study, where 43% of 122 persons with ASD also had ADHD, we found only 4% with a registered diagnosis of ADHD. This may indicate that awareness of the possible existence of ADHD not only in children and young adults but also in older adults is a recent phenomenon (Michielsen et al. 2012; Guldberg-Kjär et al. 2013). The former belief, as expressed in the DSM-IV (American Psychiatric Association 1994), that ADHD should not be diagnosed in individuals with ASD, may also provide an explanation for these low numbers of ADHD diagnoses.

In our study, the total number of individuals diagnosed with any psychotic disorder was 71, or almost 12%; this figure roughly matches the findings of Hofvander et al. (2009), while Lever and Geurts (2016) had schizophrenia as an exclusion criterion. In another sample from an adult psychiatric clinic (n = 270), 21% had been diagnosed with a psychotic disorder (Nylander et al. 2012); and in a recent population-based study, Supekar et al. (2016) found a prevalence of 18% for schizophrenia in adults 35 years or older with ASD. Thus, our results do not differ from other findings, and point toward a high prevalence of psychotic disorders in middle-aged or older individuals with ASD.

In a recent study by Lever andGeurts (2016), examining 48 individuals with ASD, 55–79 years old and with IQ > 80, 66.9% were found to have some kind of lifetime psychiatric disorder, and 44% reported symptoms consistent with a mood disorder diagnosis. Lever and Geurts also found that self-reported psychiatric symptoms were more common in this ASD group of older people than in an age-matched control group, but less common than in two comparison groups of younger people with ASD. In our sample, which is not based on self-reported lifetime symptoms but on registered diagnoses, 49.6% of the whole ASD group had some kind of psychiatric diagnosis during the period examined, and 19.6% had had a mood disorder diagnosis. Of the group (n = 345) who had no ID diagnosis registered, 69.2% had some kind of psychiatric diagnosis but only 10.8% had affective disorder diagnoses. Twelve percent had some kind of psychotic disorder diagnosis, which is the same prevalence as was found by Hofvander et al. (2009).

Of our ASD group, 381 patients (63.4%) had been in contact with specialised psychiatric care and 151 (25%) had been in-patients during the period examined. This exceeds the numbers (20% in contact with psychiatric care) reported by Axmon et al. (2016) for the larger group of older people eligible for LSS services, which in turn exceeded the 6% in the matched group from the general population. Only 15 individuals, or 11%, of the group with Asperger’s syndrome had not been in contact with psychiatric care, and 43% had been psychiatric in-patients, which may be interpreted as a sign of vulnerability in these individuals. The OR for being in contact with specialist psychiatry was almost seven times higher for people with Asperger’s syndrome compared to people with childhood autism. Also, people with Asperger’s syndrome exclusively had been in contact with substance dependency treatment clinics or forensic psychiatry, which may point to a greater risk for substance dependency and/or criminal behaviour in this intellectually more able group. Other factors raising the OR for psychiatric contact were the presence of ID, male gender and belonging to the younger rather than older group.

Other authors have found that psychotropic medication, including polypharmacy, is frequently prescribed to adults with ASD (Esbensen et al. 2009; Åkerström 2001; Buck et al. 2014; Jobski et al. 2017). In the group that we studied, 63% of patients without registered ID diagnosis and as many as 84% of those with ID in combination with ASD had been prescribed antipsychotic medication. This is in contrast to the number (12%) of individuals with diagnosed concomitant psychotic disorders but may be seen as an indication that it is common practice to treat people with ASD, especially if they also have ID, with antipsychotics as a way of managing behaviours. The effects and side effects of these treatments in adults and older adults require investigation (Näslund 2007). Also the other categories of psychotropic drugs investigated were more frequently prescribed to individuals with double diagnoses. Three or four different psychotropic drugs were prescribed to 32 and 24% of all individuals examined, respectively. Only 58 (9.6%) of all indiviuals in the study had not been prescribed any of the studied psychotropic drugs during the study period, although 50% had no psychiatric diagnosis other than ASD, with or without ID registered. Compared to other findings, e.g. those of Jobski et al. (2017), this group of older adults with ASD were very often prescribed psychotropic drugs.

We have no way of knowing to what extent the group in our study is representative for all older adults in Sweden with ASD. The group was selected according to three criteria: (1) having received LSS service in 2012, (2) having been in contact with secondary health care and (3) having been assigned an ASD diagnosis during 2002–2012. It is likely that several individuals in the studied age group had not been given any LSS service, and/or not been in contact with health care, and/or not assigned an ASD diagnosis when seeking medical care. It is also probable that some individuals with ASD have not been classified in LSS group 1, but have received services resulting from classification in one of the other two groups. However, since 87% of all LSS service users have been classified in LSS group 1 (Socialstyrelsen 2017), the number of older people with ASD in LSS groups 2 or 3 is probably very small. Our results should thus be seen as minimum numbers, and to our knowledge it is the largest group of older adults with ASD hitherto described in the world. The selection of cases on the basis of the LSS-register does “guarantee” that the individuals included had significant functional impairments, and therefore meet the basic requirements for an ASD diagnosis.

The results showed that there are older people in Sweden with ASD diagnoses who have significant needs for services, and who require psychiatric specialist services due to concomitant psychiatric disorders. It seems that the group with Asperger’s syndrome, or ASD without ID, is especially vulnerable to psychiatric disorders. There is, as a number of authors have pointed out, a lack of research concerning ASD in this age group, and it is likely that ASD is underdiagnosed and that older individuals with ASD are provided with inadequate support due to this lack of knowledge. Still, not much is known concerning which socio-economic conditions older individuals with ASD live under, which services they need or which services they actually are offered.
